# Dystonic Movement Disorder as Symptom of Catatonia in Autism Spectrum Disorder

**DOI:** 10.1155/2020/8832075

**Published:** 2020-12-31

**Authors:** Maria Pia Riccio, Rosamaria Siracusano, Ilaria d'Alessandro, Maria Marino, Carmela Bravaccio

**Affiliations:** ^1^Department of Translational Medical Sciences, Child Neuropsychiatry Unit, University Federico II of Naples, Via Sergio Pansini 5, Naples, Italy; ^2^Cognitive Psychotherapy School (S.P.C.), Naples, Italy

## Abstract

**Background:**

Catatonia is increasingly recognized as a comorbid psychiatric condition in autism spectrum disorder (ASD), but the overlap of behavioral characteristics between these disorders raises many diagnostic challenges. Moreover, recognizing symptoms in ASD patients with medium-low functioning might be difficult. Literature on this argument is poor, especially for children. *Case presentation*. We report the case of an ASD patient with low cognitive functioning, who presented a complex symptomatology, characterized by progressive regression with loss of autonomy and involuntary movements that assume “dystonic” features. Organic pathology was excluded, and catatonia, with peculiar dystonic characteristics, was diagnosed. An intervention based on elimination of stressful factors, resumption of routines, and support for parents led to the resolution of catatonic symptoms.

**Conclusions:**

The case describes the presence among the catatonic symptoms in ASD of involuntary “dystonic” movements; so far, little reported in literature; it highlights that the catatonia may present with a broad spectrum of motor abnormalities. There is still little evidence for treatment of catatonia and ASD. Our case highlights how it is equally important to take into account triggering factors when implementing a nonpharmacological treatment. So, it represents an example of diagnostic and therapeutic challenges of catatonia in ASD, especially in low functioning forms.

## 1. Introduction

Catatonia is increasingly recognized as a comorbid psychiatric disorder in autism spectrum disorder (ASD), but the overlap of behavioral characteristics between these disorders raises many diagnostic challenges. Moreover, recognizing symptoms in ASD patients with medium-low functioning is difficult. Literature on this argument is poor, especially for children.

The described case is particularly interesting for the presence of involuntary movements that assume “dystonic” characteristics, among the catatonia symptoms. This case represents an example of diagnostic and therapeutic challenges of catatonia in ASD; we therefore believe it will make a contribution to correctly identifying comorbidity diagnoses in children and adolescents with ASD.

## 2. Case Presentation

We report the case of a patient with autism spectrum disorder (ASD) who presented a complex symptomatology and required hospitalization and careful observation to arrive at a correct diagnosis.

A is a 7-year-old boy diagnosed with ASD; he presents low cognitive functioning and moderate level of autistic symptomatology. Previously, genetic investigation (karyotype, CGH-array), metabolic screening, and brain magnetic resonance imaging (MRI) were performed, with negative results. Electroencephalogram (EEG) during sleep showed nonspecific atypia, bilaterally on the central-temporal regions. A attends the first year of primary school with support of a teacher, practices psychomotor, speech, and music therapy. His level of language consisted of simple sentences, used above all for requests. He used some descriptive gesture; pointing was present as request and declarative function. Personal autonomy in the acts of daily life was quite sufficient: he used the bathroom and ate independently. There were frequent stereotyped movements (mannerisms and flickering). Six months before he visits the hospital, he presented a period of behavioral control disorder, with an increase in hyperactivity and agitation in concomitance with the separation of his parents. For this reason, the hours of therapies have been increased, the figures in the rehabilitation setting have been changed, and highly structured behavioral techniques have been introduced (e.g., use of operating conditioning). A showed a progressive loss of verbal and motor skills and loss of previous interests. At the time of admission, A had no initiative, mimicry characterized by a “smiling” facial expression, not congruent to the emotional state, and absence of response to questions and requests. He presented loss of autonomy with the need for continuous assistance (to be fed during meals, exhorted, and helped to go to bathroom). At the neurological examination, focal signs were absent, but A presented a fixed posture and involuntary movements, typically patterned as twisting, involved upper and lower limbs. According to clinical criteria [1], these involuntary movements seemed to assume characteristics of dystonic features. Detailing, movement distribution was multifocal, and phenomenology was persistent during the day and not linked to specific triggers; moreover, no others neurological manifestations occurred. At beginning, an isolated dystonia was hypothesized. A complete evaluation was assessed in order to consider possible etiology. The patient underwent blood tests to evaluate the endocrinological, metabolic, coagulative, and autoantibody profile, which result all within normal range. He repeated EEG confirming the result of the previous exam. The ophthalmologic consultation and fundus examination were normal. Neuroradiological examination with brain MRI resulted without significant pathological findings. An organic pathology (metabolic, genetic, or acquired conditions) underlying the disorder was excluded. Considering clinical, instrumental, and anamnestic information, it was hypothesized that the abnormal dystonic movements and postures of the patient might be a phenomenology of a catatonia disorder associated with ASD. Parents refused drug therapy. Treatment was modified with reduction of hours, return of the previous operators, and beginning of a parent training program. In particular, the introduced highly structured behavioral techniques were removed, in favor of a work aimed at supporting the functions of intersubjectivity (i.e., support ludic interest and functional use of objects, improve social playful exchange, and communication functions). Family routines, changed due to parental separation, were resumed. The case was monitored by telephone on a weekly basis. After a month, A showed a slight improvement, involuntary movements were no longer present, the motor picture was more fluid with reduction of fixed posture, and he had started to say a few words. In the following months, he improved gradually, with recovery of skills and autonomy. After four months, the previous level of motor and daily functioning was resumed, confirming that dystonic phenomenology was a manifestation of catatonia.

## 3. Discussion

ASD has a high rate of psychiatric comorbidity, and catatonia is increasingly recognized as a comorbid disorder [2]; its prevalence in ASD adolescents and young adults varies from 12 to 20% [3–5]. No symptoms are pathognomonic for catatonia and can vary widely between patients and over time [6]. Diagnosis of catatonia is clinical; according to DSM-5 [7], regardless of the associated disorder, it is based on the presence of at least 3 out of 12 criteria: stupor, catalepsy, wax flexibility, mutism, negativism, fixed posture, mannerism, stereotypy, agitation, and presence of grimace. There is therefore an overlap of behavioral characteristics between catatonia and ASD that raises many diagnostic challenges [8, 9]. Some studies have hypothesized that early onset of catatonia may actually be a variant of the autism spectrum disorder [10, 11] and furthermore, that some patients with early-onset catatonia may be misdiagnosed as ASD. It is our opinion that, while the diagnostic criteria of ASD are precise enough to allow a differential diagnosis between these conditions early on, it is far more challenging to diagnose them in comorbidity, especially since recognizing symptoms in a patient with ASD with medium-low functioning is difficult.

The case described is particularly interesting for the presence among the catatonic symptoms of fixed posture and motor overflow (involuntary movements typically patterned as twisting, involved upper and lower limbs, multifocal, persistent during the day, and not linked to specific triggers) that assumed “dystonic” characteristics. Numerous studies report the appearance or increase of involuntary movements, the majority of which are simply described as stereotypies. So, it is possible that the described symptomatology is an uncommon manifestation, little reported in literature. A high rate of motor anomalies is described in the ASD and catatonia association: one odd gait (90%), odd, stiff postures (63.3%), freezing (56.7%), impulsivity (53.3%), inability to stop actions (23.3%), and excited behavior (23.3%) [4]. Overall, Billstedt et al. (2005) report “clinically diagnosed catatonia with severe motor initiation problems” as outcome in 12% of 120 ASD adolescents. In particular, patients were reported to have very awkward gait movements, according with the high rate of posturing in patient with catatonia. It is therefore conceivable that in ASD who also present catatonia, the motor anomalies writable to the latter and often counted as stereotypies show varied facets and need a careful evaluation.

In the case described, parents report, before the full-blown symptoms, a phase characterized by an increase in hyperactivity. Some studies [12] point out that the alternation of agitation and stupor is almost pathognomonic of catatonia. We are not sure that onset of catatonia in our patient was characterized by excitement, but it is certainly important to report this data because, especially in cases of poorly verbal and low functioning ASD, behavioral changes are often the only sign of onset of comorbid psychiatric pathologies. This often results in the absence or delay of psychiatric diagnoses in ASD patients. Moreover, the onset seems to be triggered by an interruption of daily routines and by exposure to stress (separation of parents). It is reported that traumatic or anxiety-provoking life events precede the onset of catatonia, suggesting a role in psychogenic factors [4]. Furthermore, we believe that a significant contribution to the worsening of the clinical picture has been determined by the change in psychoeducational therapy.

Numerous clinical studies indicate a multimodal approach for the treatment (psychological interventions, high doses of lorazepam, bilateral electroconvulsive therapy-ECT); treatments are used in a graded way, according to catatonic severity and response to previous ones; behavioral treatments seem to have some positive outcomes, although no cases with complete resolution of symptoms are reported only with nonpharmacological treatment [2, 6, 8, 13]. In our case, parents did not give consent for pharmacotherapy.

Psychoeducation for caregivers, reducing stress, encouraging engagement in activities, use of prompting, and maintaining structure/routine are all suggested strategies for behavioral interventions [1]. So, our approach is in line with the few studies described in literature: the intervention based on elimination of stressful factors, resumption of routines, support for parents, and invitation to activities he likes, in this case led to the resolution of catatonic symptoms.

This case represents an example of diagnostic and therapeutic challenges of catatonia in ASD; it demonstrates a clear catatonic deterioration from baseline, but at the same time, we describe some symptoms so far little reported in literature, emphasizing the diversity with which motor anomalies can occur in this association. We therefore believe it will make a contribution to correctly identifying comorbidity diagnoses in children and adolescents with ASD. There is still little evidence for treatment of catatonia and ASD. In this sense, our case highlights how it is equally important to take into account triggering factors when implementing a nonpharmacological treatment.

## Data Availability

The datasets used and/or analyzed during the current study are available from the corresponding author on reasonable request.
